# The role of anillin/Mid1p during medial division and cytokinesis: from fission yeast to cancer cells

**DOI:** 10.1080/15384101.2022.2147655

**Published:** 2022-11-25

**Authors:** Imane M. Rezig, Wandiahyel G. Yaduma, Gwyn W. Gould, Christopher J. McInerny

**Affiliations:** a School of Molecular Biosciences, College of Medical, Veterinary and Life Sciences, Davidson Building, University of Glasgow, Glasgow, UK; b Department of Chemistry, School of Sciences, Adamawa State College of Education Hong, Nigeria; c Strathclyde Institute of Pharmacy and Biomedical Sciences, University of Strathclyde, Glasgow, UK

**Keywords:** Mid1p, anillin, fission yeast, cytokinesis

## Abstract

Cytokinesis is the final stage of cell division cycle when cellular constituents are separated to produce two daughter cells. This process is driven by the formation and constriction of a contractile ring. Progression of these events is controlled by mechanisms and proteins that are evolutionary conserved in eukaryotes from fungi to humans. Genetic and molecular studies in different model organisms identified essential cytokinesis genes, with several conserved proteins, including the anillin/Mid1p proteins, constituting the core cytokinetic machinery. The fission yeast *Schizosaccharomyces pombe* represents a well-established model organism to study eukaryotic cell cycle regulation. Cytokinesis in fission yeast and mammalian cells depends on the placement, assembly, maturation, and constriction of a medially located actin-myosin contractile ring (ACR). Here, we review aspects of the ACR assembly and cytokinesis process in fission yeast and consider the regulation of such events in mammalian cells. First, we briefly describe the role of anillin during mammalian ACR assembly and cytokinesis. Second, we describe different aspects of the anillin-like protein Mid1p regulation during the *S. pombe* cell cycle, including its structure, function, and phospho-regulation. Third, we briefly discuss Mid1pindependent ACR assembly in *S. pombe*. Fourth, we highlight emerging studies demonstrating the roles of anillin in human tumourigenesis introducing anillin as a potential drug target for cancer treatment. Collectively, we provide an overview of the current understanding of medial division and cytokinesis in *S. pombe* and suggest the implications of these observations in other eukaryotic organisms, including humans.

## 1. Introduction

### 1.1 Anillin-based contractile ring drives cytokinesis in mammalian cells

Cytokinesis initiation requires establishment of a medial division plane, the assembly of an actin-myosin contractile ring (ACR), and the ingression of a cleavage furrow, (*see* [1]for review). The scaffold protein anillin has a pivotal role in organizing the cytokinetic machinery and linking the ACR to the plasma membrane [2].

**Figure 1.**
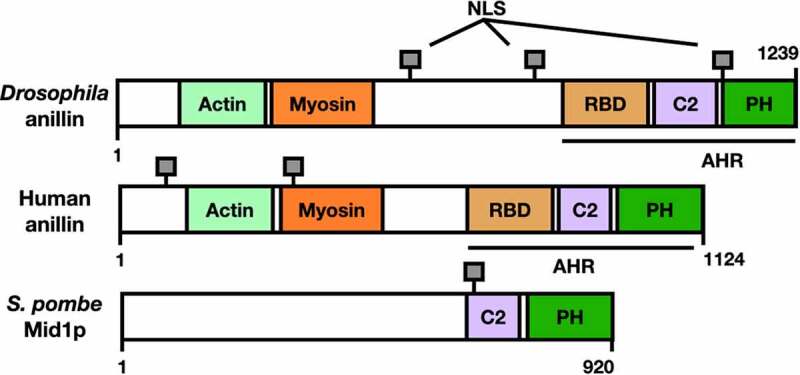
Anillin-related proteins in different systems show structural homology. The different characterized or putative domains are colour coded. RBD: Rho binding domain, C2: cryptic domain, PH: pleckstrin homology domain. Anillin homology region (AHR) and nuclear localization signals (NLS) are indicated. See main text for references.

Anillin was first identified in the fruit fly *Drosophila melanogaster* in 1989 as an Factin-binding protein [3] (Figure 1); homologues of this protein were then characterized in all eukaryotes including the fission yeast Mid1p (also called Dmf1p) [4] [5] (Figure 1), and the human anillin [6] (Figure 1). These anillin-related proteins share a general structure that is conserved in metazoans [7]. The N-terminal region of human anillin includes binding sites for F-actin and myosin; such interactions with anillin are required for organization of the ACR [8]. The C-terminal region of human anillin contains three main domains: a Rho binding domain (RBD), a cryptic domain (C2), and a pleckstrin homology domain (PH); these domains promote efficient recruitment to the plasma membrane [2].

### 1.2 Fission yeast as a model organism to study mammalian cytokinesis

The fission yeast *Schizosaccharomyces pombe* is a well-established model organism used for studying the eukaryotic cell cycle due to its short doubling time, simple organization, and tractable genetics [9]. The rod-shaped *S. pombe* cells grow by elongation at cell tips during interphase, then stop growth to divide through the assembly of a medial actin-myosin contractile ring (ACR) composed of actin filaments (F-actin) and type-II myosin (Myo2) [10, 11]. Medial formation of the ACR requires functions of the scaffold protein anillin/Mid1p [12].

Over the past decade, studies with *S. pombe* have led to a comprehensive understanding of Mid1p’s function during ACR assembly and cytokinesis [12–16] [17,18] [19].

In this review, we describe aspects of ACR assembly and cytokinesis regulation in fission yeast and the regulation of such events in mammalian cells. First, we briefly describe the role of anillin during mammalian ACR assembly and cytokinesis (Section 2). Second, we focus on the different aspects of Mid1p regulation the *S. pombe* cell cycle, including Mid1p protein structure, biological functions, and phospho-regulation (Section 3). Third, we describe recent findings suggesting a Mid1p-independent ACR assembly mechanism during the *S. pombe* cell cycle (Section 4). Finally, we end with discussion of exciting recent studies that offer new insight into the emerging role of anillin in human tumourigenesis which introduce it as potential drug target for cancer treatments (Section 5).

## 2. Anillin-dependent assembly of ACR in mammalian cells

### 2.1 Anillin links the ACR to the plasma membrane during mammalian cell cycle

**Figure 2.**
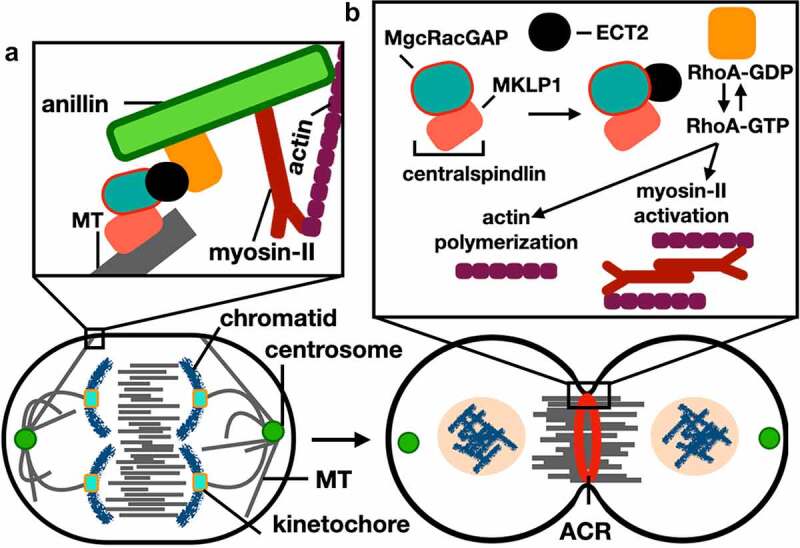
Cleavage furrow formation and ingression during cytokinesis in mammalian cells. Schematic representation of cells in anaphase (left) and telophase (right). (a) Anillin functions in connecting the spindle MT with the ACR through interactions with RhoA (*see* (b) for labels). (b) RhoA GTP-dependent activity is induced by interactions with the MgcRacgap and ECT2, after which leads to polymerization of actin filaments and induction of the phosphorylation-dependent myosin-II activation; these events lead to the formation and ingression of the cleavage furrow through interactions between myosin heads and actin filaments. *See* main text for references.

In mammalian cells, determining the site of division and the formation of a medial ACR involves activation of the small GTPase RhoA pathway [20]. Rho GTPases are regulated through switching their GDP/GTP status, in which activation is triggered by the Rho-specific guanine nucleotide exchange factors (RhoGEFs) through stimulating disassociation of the tightly bound GDP (*see* [21]for review). Exchange factor epithelial cell transforming sequence (ECT2), the direct upstream activator of RhoA, binds and activates GTP-bound RhoA [22]. Activation and recruitment of ECT2 is regulated by the centralspindlin complex during anaphase, with this complex being a heterotetramer composed of two dimers: Male Germ Cell (MgcRacGAP) and the Mitotic Kinesin Like Protein (MKLP1) [23] (Figure 2).

Once RhoA is activated, anillin is recruited to the site of division and integrates the RhoA signaling pathway with ACR formation [24]. Anillin has a direct role in connecting the ACR to the spindle microtubules and the plasma membrane to stabilize the cleavage furrow [8,25]. During abscission the ingressed cleavage furrow, a thin plasma membrane-based intracellular bridge (ICB) composed of bundles of microtubules, connects the two daughter cells. Recently, anillin was found to be involved in the biogenesis of the ICB by interacting with septin filaments (SEPT9) and the CIN85 complex to elongate and thin the ICB, whereby CIN85 connects the N-terminal domain of anillin to SEPT9 promoting active elongation of the ICB [25].

Anillin leaves the ICB prior to the last step of abscission [25] and, subsequently, cleavage of the ICB by the Endosomal Sorting Complex Required for Transport (ESCRT) proteins during abscission leads to the final separations of cells [26]. (Figure 2) describes the role of anillin during mammalian cytokinesis, *see* **Table S1** for *S. pombe* homologue proteins.

## 3. Mid1p-dependent assembly of ACR in *S. pombe* cells

### 3.1 *Medial positioning of the ACR in* S. pombe *is linked to the position of the nucleus and Mid1p localization to assemble cortical nodes*

The nucleus in *S. pombe* cells is medially positioned. This is achieved through the microtubules (MTs) organizing center which form the MTs into antiparallel bundles along the long axis of the cell during interphase [27,28]. It was suggested by [29]that medial positioning is preserved in *S. pombe* nuclei due to the opposing pushing forces generated by the interphase MTs located on both cell ends, and that growth of MTs at cell tips pushes the nucleus. Furthermore, computer modeling revealed that these pushing forces are balanced to create a mechanism for medial positioning of the nucleus [29]. Based on a significant body of evidence, it is hypothesized that the position of the nucleus is critical for medial ACR positioning in *S. pombe*.

Daga and Chang [30] tested this hypothesis and demonstrated that moving the nucleus away from the cell center in *S. pombe* influences the position of ACR assembly during early mitosis. Such nuclear displacement experiments resulted in the formation of multiple ACRs.

Mid1p is non-essential for viability in *S. pombe*, however, the absence of Mid1p leads to cytokinesis failure at higher temperatures [4]. Since the identification and characterization of Mid1p, its localization pattern throughout the cell cycle of *S. pombe* has been extensively studied [12–16] [17,18] [19].

**Figure 3.**
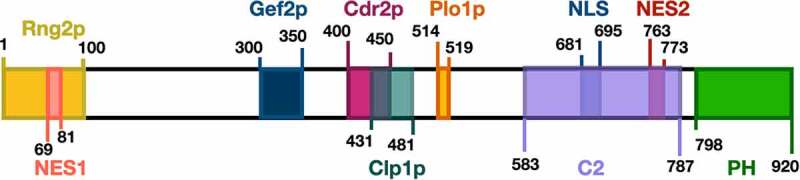
Schematic of *S. pombe* Mid1p domain organization and the binding sites of proteins involved in cytokinesis. (A) Rng2p [13], Gef2p [31], Cdr2p [14], Clp1p [32] and Plo1p [13] binding sites in addition to the nuclear export sequence NES, C2 domain, nuclear localization sequence NLS, and pleckstrin homology domain PH [12], are shown in different colours.

Initially, Mid1p localizes to the nucleus and shuttles between the nucleus and cell cortex using nuclear localization (NLS, Figure 3) and nuclear export (NES, Figure 3) sequences during interphase and early mitosis [12]. Mid1p is then phosphorylated by the polo-like kinase Plo1p which targets the export of Mid1p from the nucleus to the cytoplasm [13], where it recruits several other proteins to assemble two types of nodes [33].

**Figure 4.**
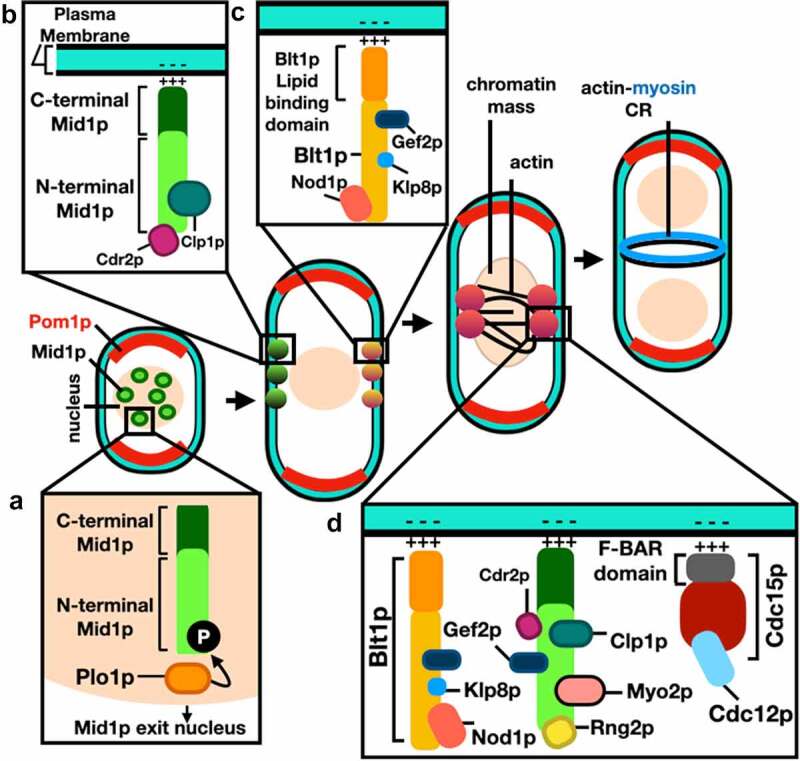
Mid1p-dependent actin-myosin contractile ring (ACR) assembly in *S. pombe*. (a) Phosphorylation of Mid1p by Plo1p kinase triggers its release from the nucleus. (b) Type-I interphase nodes form by sequential recruitment of Mid1p, Cdr1p and Cdr2p, they interact with the cell membrane through the PH and C2 domains of Mid1p. (c) Type-II interphase nodes form by sequential recruitment of Gef2p, Blt1p, Klp8p and Nod1p, they interact through the membrane binding domain of Blt1p. (d) Cytokinesis nodes form when Type-II interphase nodes are captured by Type-I nodes after they migrate to the medial cortex, and upon the onset of mitosis, Myo2p, Rng2p, Cdc12p and Cdc15p are recruited to interphase nodes, cytokinesis nodes then condense into the actin-myosin contractile ring. *See* main text and Table S1 for references and mammalian homologue proteins. .

Type-I interphase nodes are composed of Mid1p and the Cdr1p and Cdr2p kinases [34]. Type-II interphase nodes form from components of the previous division disassembled ACR, including Gef2p, Blt1p, Klp8p, and Nod1p [33]. Type-I nodes interact with the cell membrane through the pleckstrin homology (PH) domain and the cryptic (C2) domains of Mid1p [2], while Type-II nodes interact through the phospholipid-binding protein Blt1p [35]. Upon the onset of mitosis Myo2p, Rlc1p, Cdc4p, Rng2p, Cdc12p, and Cdc15p are recruited to interphase nodes to form the cytokinesis nodes [36,37]. Cdc12p is responsible for the polymerization of actin filaments and, at this stage, interactions between Myo2p and the polymerized actin filaments lead to cytokinesis nodes condensation assembly of the ACR [38]. Schematic representations of Mid1p domain organization-binding sites of cytokinesis proteins, and Mid1p-dependent ACR assembly mechanism are shown in Figures 3 and 4, respectively.

The number and composition of cytokinesis nodes has been extensively studied in fission yeast [19–33] [34–36] [37,38] [39–41]. However, some studies underestimated the number of cytokinesis nodes due to limitations in imaging techniques and the lack of a 3D reconstitution approach. For example, closely spaced nodes cannot be resolved using conventional confocal microscopy.

Using super-resolution single-molecule localization microscopy [42] showed that cytokinesis nodes are uniform in size and composition. A recent study by Sayyad and Pollard [43] used a 3D reconstitution approach using Airyscan fluorescence imaging in live *S. pombe* cells to count the total number of single cytokinesis nodes. Using Blt1p as a cytokinesis marker in wild-type cells, 190 cytokinesis nodes were detected at the cell equator during early mitosis. Furthermore, 85% of Blt1p-mEGFP in these nodes is incorporated into the ACR during early mitosis [43].

### 3.2 *Medial positioning of the ACR in* S. pombe *is mediated by Pom1p kinase*

The dual-specificity tyrosine-regulated kinase (DYRK) Pom1p plays a regulatory role in medial positioning of the ACR in *S. pombe* through a “tip occlusion” inhibitory mechanism [44], whereby a Pom1p-based gradient emanating from the cell tips act as a negative signal to regulate division plane placement. Additionally, reversible binding of Pom1p to the plasma membrane is affected by its phosphorylation status [45].

The polarity determinant Tea4p phosphatase is deposited at the cell tips through interactions with microtubules [46]. Tea4p recruits Pom1p to the cell cortex through de-phosphorylation, and this triggers lateral movement of Pom1p at the plasma membrane through Pom1’s lipid-binding region. Pom1p is then released into the cytoplasm through auto-phosphorylating its lipid-binding region [44]. [47] confirmed *in vitro* and *in vivo* intermolecular auto-phosphorylation of Pom1p; furthermore, Pom1p gradient decay length showed a strong negative correlation with Pom1p amplitude, suggesting that this correlation results from a buffering mechanism on the decay length.

[48] utilized super resolution microscopy to track individual Pom1p molecules inside *S. pombe* cells. They found that Pom1p travels between clusters in a “hopping” manner to move from the cell tip toward the medial region of the cell, with these clusters creating the gradient. Additionally, they confirmed Pom1p distribution at the plasma membrane through the cycle of Pom1p gradient phosphorylation and de-phosphorylation events [49]. revealed that the PP2C phosphatase Ptc1p dephosphorylates Pom1p *in vitro*, and both proteins are able to form complexes *in vivo*. They propose a scenario where Ptc1p influences Pom1p distribution through reversing its phosphorylation status. Another recent study by [50] looked at the status of Pom1p in the absence of Mid1p. They found that Pom1p prevents division at cell tips even in the absence or mis-localization of Mid1p. Their results also revealed that the phosphorylation of the mitotic inducer Cdc15p by Pom1p kinase disrupts its membrane-binding ability; this disruption inhibits Cdc15p’s scaffolding function during cytokinesis. Such studies emphasize a need to understand the control of phosphorylation dynamics in Mid1p, as discussed in (Section 3.4).

### 3.3 The structure and molecular function of Mid1p

Saha and Pollard [18] investigated the biological functions of Mid1p domains during the *S. pombe* cell cycle and found that residues (1–149) of Mid1p are essential for the correct orientation and positioning of septa. Furthermore, the same residues (1–149) are required but not sufficient for the localization of full-length Mid1p to cortical nodes. However, residues (1–452) facilitate Mid1p functions including localization and concentration in cortical nodes during mitosis, while residues (1–578) are required for the assembly of several node components including Myo2p and Cdc15, and residues (579–797) resemble the insoluble domain of Mid1p and facilitate condensation of nodes into the ACR.

**Figure 5.**
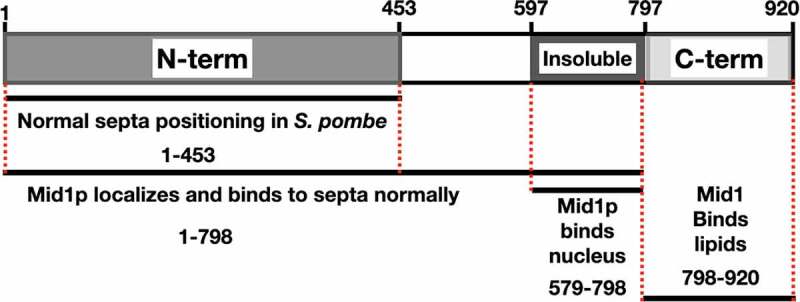
A schematic of Mid1p domains and their role during mitosis and cytokinesis in S. pombe. (a) Mid1p residues 1-453 resemble the N-terminal domain. (b) Mid1p residues 579-797 resemble the Insoluble domain. (c) Mid1p residues 798-920 resemble the C-terminal PH domain. Red broken lines represent Mid1p domain boundaries. Black lines represent fragments of Mid1p sufficient for the role denoted below each line. Figure adapted from Saha and Pollard (2012).

Residues (798–920) of Mid1p contain the C-terminal PH domain [5,18]. The structure of Mid1p has two membraneanchoring elements, the C2 lipid-binding domain and the PH domain [2]. The Mid1p-N452 domain, composed of the Mid1p N-terminal residues (1452), is intrinsically disordered, and this flexible nature may facilitate the export of Mid1p from the nucleus during early mitosis [15]. Interestingly, this domain of Mid1p contains multiple residues that are phosphorylated when expressed in insect cells. This appears to regulate self-association of Mid1p-N452. Present models suggest that phosphorylation (e.g. by Plo1p - *see* Section 3.4) could control Mid1p export from the nucleus by “solubilizing” the protein, as non-phosphorylated Mid1p-N452 has increased tendency to aggregate [15]. Of note, this domain does not interact with Myo2 [15]. A schematic representation of the Mid1p structural domains is shown in (Figure 5).

It is worth noting that both mammalian anillin and *S. pombe* Mid1p share functional similarities, hence both are multi-domain scaffolding proteins and bridge the cell cortex with the ACR during mammalian [51] and *S. pombe* [33] cytokinesis, respectively. Furthermore, functional analysis of mammalian anillin and *S. pombe* Mid1p showed that both proteins have cryptic membrane associating elements and bind to membrane lipids through a C2 cryptic domain [2]. The following section describes Mid1p molecular structure and phospho-regulation during *S. pombe* cell cycle.

### 3.4 Phospho-regulation of Mid1p

[15]investigated the phosphorylation of Mid1p N-terminal region including residues (1–452): Mid1p-N452. They used two methods: the disorder-enhanced phosphorylation predictor (DISPHOS) tool to predict phosphorylation sites, and matrix-assisted laser desorption/ionization (MALDI) mass spectrometry to identify phosphorylated residues. Such analyses confirmed Mid1pN452 phosphorylation by six of the nine consensus Sid2p phosphorylation sites [52], three of four minimal consensus Cdk1p phosphorylation sites [53], and one of the eight consensus Plo1p phosphorylation sites [54]. Phospho-regulation of Mid1p by various kinases is discussed below.

Early studies by [55]described a physical interaction between the *S. pombe* polo-like kinase Plo1p and Mid1p proteins, and that this interaction was required for the correct localization of Mid1p to the ACR. It was later confirmed by [13]that phosphorylation of residues within the first 100 amino acids of the N-terminal region of Mid1p by Plo1p triggers Mid1p release from the nucleus and promotes the association of Mid1p with interphase nodes leading to mitotic entry. Such phosphorylation also facilitates Myo2p recruitment to medial cortical nodes.

While Mid1p has a regulatory role of ACR medial assembly, the NDR-family kinase Sid2p has a controlling role during the later stages of cytokinesis to promote ring constriction and septation leading to completion of cell division. However, Mid1p departs from the site of division at ACR constriction onset [5]; this event is concurrent with the translocation of Sid2p from spindle pole bodies (SPBs) to the ACR [56]. Sid2p consensus phosphorylation motifs are found in the Mid1p amino acid sequence [52].

[19]found that a Mid1p phospho-deficient mutant that cannot be phosphorylated by Sid2p kinase remains attached to the plasma membrane throughout cytokinesis. Furthermore, after completion of cell division this mutant over-accumulates in interphase nodes and leads to early recruitment of ACR proteins to interphase nodes. This study also confirmed the phosphorylation of Mid1p by Sid2p on residues within the N-terminal domain (1–578); additionally, it provided evidence that removal of Mid1p from the cell cortex is driven by this phosphorylation event.

[16]revealed that Cdc42-activated polarity kinase (Pak1p) is localized to the assembling ACR and maintains this localization during septation. In this study, a large-scale phospho-proteomic screen identified Mid1p and Cdc15p as Pak1p substrates. Disturbing the Pak1p/Mid1p signaling pathway produced defective and misplaced ACRs; however, such defective phenotypes are rescued by synthetic tethering of Mid1p to cortical nodes. Therefore, it is suggested that Pak1p phosphorylation of the N-terminal region of Mid1p promotes its association with interphase nodes.

**Figure 6.**
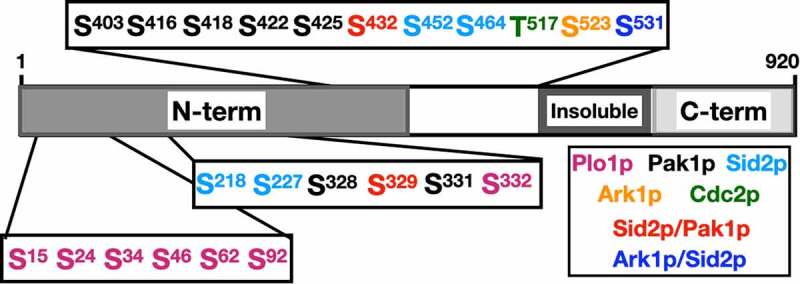
Schematic of *S. pombe* Mid1p phospho-regulation. Phosphorylation sites of Mid1p - *see* Figure 5 legend for Mid1p domain structure - by Plo1p [13, 17], Cdc2p [13], Pak1p [16], Ark1p [17], and Sid2p [19]. Please *see* Table S2 for description of each phosphorylation event.

Interestingly, the N-terminal region of Mid1p is phospho-regulated by three kinases. Phosphorylation by Plo1p promotes Mid1p nuclear export and the onset of mitosis [13], phosphorylation by Pak1p promotes Mid1p association with interphase nodes [16]; and phosphorylation by Sid2p promotes Mid1p removal from the cell cortex [19]. Phospho-regulation of Mid1p is schematically represented in Figure 6.

Recent work tested the genetic interactions *in S. pombe* between three classes of genes: *mid1*, the ESCRT *vps4*, and the aurora kinase *ark1*. Genetic interactions were detected between the *mid1* gene and both *vps4* and *ark1* genes; such interactions suggest a link between the regulation of Mid1p’s function by both Vps4p and Ark1p [17,57]. Furthermore, it was found that Vps4p physically interacts with the C-terminal region of Mid1p, with this interaction important for the localization of Mid1p in cells. It appears that the function of Mid1p is regulated by associating with Vps4p, with this association directly or indirectly involving the Mid1p PH domain cell cortex anchorage to regulate Mid1p-dependent node cortical attachment to promote medial division [17].

Mid1p phospho-regulation was examined by combining three approaches: *in vitro* phosphorylation studies, tandem mass spectrometry (nLC-MS/MS) analysis and interrogating published *S. pombe* global proteomic data. Such approaches identified several amino acid residues as potential phospho-acceptor sites in Mid1p by aurora and polo-like kinases (Figure 6), with S332, S523, and S531 required for the function of Mid1p in *S. pombe* [17].

Despite the major role of Mid1p is cytokinesis, recent studies suggest that Mid1p is dispensable for the organization of cytokinesis proteins into nodes [58]. The next section therefore discusses Mid1p-independent ACR assembly mechanisms.

## 4. Mid1p-independent assembly of ACR during the *S. pombe* cell cycle

### 4.1 *Mid1p-independent molecular organization of nodes during the* S. pombe *cell cycle*

In *S. pombe*, a chromosomal deletion of the *mid1* gene (*mid1*Δ) causes dramatic defects in septation phenotypes resulting in branched and multi-septated cells; furthermore, ACR assembly is delayed in *mid1*Δ cells [5,14,59]. This confirms the requirement of Mid1p function for correct division plane positioning. It is known that Mid1p recruits Rng2p to the division site, which results in the accumulation of ACR components [60]. However, is Mid1p solely responsible for regulating the ACR position?

To address this question [61], performed a “rewiring” experiment and examined the localization of several proteins in reconstituted cells, which are *S. pombe* cells artificially made to divide medially in the absence of Mid1p. Medial division in these *mid1* mutants was restored by artificial targeting of Rng2p, Cdc12p, and Myo2p to the division site. However, in these cells the ACR assembles late during anaphase suggesting that an interaction of Mid1p with one or more ACR proteins is required for ACR assembly during early mitosis. Assembly of the ACR is a complex process. Fortunately, there is a growing understanding of ACR protein composition, mechanism of assembly, and its function; but what is molecular structure of the ACR?

To answer this question [62]used super-resolution microscopy and fluorescence resonance energy transfer (FRET) to examine 29 ACR protein components and determined their spatial organization relative to the plasma membrane. This allowed the classification of ACR protein components into three layers: a proximal layer (0–0.8 nm) composed of scaffold proteins such as Mid1p and Cdc15p; an intermediate layer (80–160 nm) composed of a network of cytokinesis accessory proteins such as Rng2p; and a distal layer (160–350 nm) composed of Factin, the motor domain of myosin. Although prior knowledge indicates when proteins are recruited to the ACR, with the three layers of proteins corresponding to the order of assembly of ACR components, additional studies are required to reveal the spatial organization of signaling components of the ACR [33, 36] [37,42].

A recent study by [58]investigated the molecular organization of four cytokinesis proteins, Rng2p, Myo2p, Cdc12p, and Cdc15p, in the absence of Mid1p. They found that ACRs with the ability to constrict assemble from loops of strands composed of actin filaments and cytokinesis proteins in *mid1*Δ cells. Additionally, nodes were observed in the strands of these cells confirming that Mid1p is unnecessary for the organization of cytokinesis nodes components. Two types of strands were identified in *mid1*Δ cells, nascent strands with nodes organized into short-linked strings, and enduring strands with nodes aligning onto long strands across the cell length. These data suggest that Mid1p is dispensable for ACR positioning and organization of cytokinesis into nodes.

## 5. Cytokinesis, anillin, and cancer

### 5.1 The function of anillin in the eukaryotic cell cycle and its role in tumourigenesis

In mammalian cells, positive signals generated by the central spindle with anillin connecting the ACR to the cell cortex ensure medial placement of the ACR [51]. The scaffolding protein anillin is essential for cytokinesis regulation, and its inhibition results in cytokinesis failure and cell multi-nucleation [6]. Anillin is composed of two functional parts: the N-terminal region, triggering actin polymerization and myosin-II activation leading to the assembly of a stable ACR [63]; and the C- terminal region, associating with RhoA, septins, and PI(2,4)P_2_ connecting the ACR to the cell cortex [2,64].

Anillin is a substrate for mitotic kinases and its recruitment to the equatorial membrane is regulated by phosphorylation. Kim and colleagues identified phosphorylation of a single residue, S635, as a key determinant mediating cytokinesis regulating anillin’s recruitment to the equatorial cortex and mediating stabilization of the cleavage furrow [65].

Following its discovery, anillin was studied mainly by cell and developmental biologists to discover the mechanisms of cell division, without being associated with human diseases. However, numerous recent studies suggest that anillin is involved in tumorigenesis in various types of cells. For example [66], demonstrated that anillin is overexpressed in human gastric cancer (GC) tissues and that depletion of anillin in these tissues inhibits the proliferation of GC cells. Additionally, a recent study by Xiao and colleagues (2020) investigated the regulation mechanism of anillin in human hepatocellular carcinoma (HCC). They found that anillin had a significant facilitating effect on cell proliferation *in vitro* and induced remarkable HCC tumor growth *in vivo*. The transcription factor SRY-Box transcription factor 4 (SOX4) had an increase in expression profile which correlates with anillin; it also interacts with specific DNA sequences in the anillin gene promoter region. The microRNA miR-138 was identified as an upstream regulator of SOX4, and overexpression of anillin was induced by a potential axis of both transcription elements: SOX4 and miR-138 [67]. Overall, similar results from studies in HCC suggest a molecular mechanism with which anillin might induce tumourigenesis [68,69]. Therefore, targeting anillin and/or its upstream regulators is a potential innovative strategy for HCC treatment.

Broadly, similar results were observed in breast cancer cell lines, where knocking down anillin significantly reduces the migration of breast cancer cells [70]. Similarly, transiently knocking down anillin protein in breast cancer cells increased the number of senescent cells, with cells accumulating in the G2/M phase of the cell cycle with effects on cell morphology including poly-nucleated cells [71]; such effects are consistent with the role of anillin during cytokinesis. Additionally, anillin is markedly overexpressed in breast cancer cells [72; 73]. A mechanism for anillin function to induce tumor activity was proposed by [73]. They suggested that such tumor-promoting activity involves transcriptional re-programming of breast cancer cells affecting their self-renewal and differentiation properties. Whatever the explanation, it is clear that the mis-regulation of anillin in human cells is associated with multiple forms of tumorigenesis and cancer.

## 6. Concluding remarks

How eukaryotic cells establish medial division to complete duplication and division remains a fascinating and complex question. Studies with fission yeast have revealed important aspects of medial division including proteins participating during cytokinesis, mechanisms of division site specification, and medial ACR assembly. The scaffold proteins anillin and Mid1p play important roles during mammalian and fission yeast cell cycle, respectively. In this review, we address important questions on how the current understanding of fission yeast cytokinesis can be applied to understand the regulation of cytokinesis in mammalian cells.

Phospho-regulation of Mid1p is required to regulate *S. pombe* cell cycle events starting with Mid1p release from the nucleus to promote the association of Mid1p with interphase nodes leading to mitotic entry [13], then Mid1p association with interphase nodes [16], and finally the removal of Mid1p from the cell cortex during the later stages of cytokinesis [19]. Mid1p is a major component of cytokinesis nodes serving as precursors of the ACR during cytokinesis. Recent advances in microscopic methods allowed the dissection of the structural organization of such assemblies revealing the relationship of cytokinesis nodes and cell size [43].

Cytokinesis failure can occur due to multiple mechanisms, including altered expression of proteins that regulate cytokinesis initiation or progression [74]. Attention is being drawn toward the use of cell cycle regulators of cytokinesis as biomarkers of several cancer types, and recent reviews describe the roles of anillin [75], aurora kinases [76], and polo-like kinases [77] in tumourigenesis. Furthermore, anillin and these mitotic kinases have further cell cycle roles beyond controlling cytokinesis, which may be also related to tumorigenesis, and so could be important potential drug targets in future cancer treatments.^78-85^

## Supplementary Material

Supplemental Material

## Data Availability

The authors confirm that the data used in this review paper are available within the cited references.
