# Supplementary material for: The role of anillin/Mid1p during medial division and cytokinesis: from fission yeast to cancer cells
Source: Cell Cycle. 2022 Nov 25;22(6):633–44. doi: 10.1080/15384101.2022.2147655 (PMC9980708; doi:10.1080/15384101.2022.2147655)
Supplement: Supplemental Material [file KCCY_A_2147655_SM8627.docx]

# Supplements

Table S1: Description of the *S. pombe* genes discussed in this review and their mammalian orthologues (https://www.pombase.org).

| ***S. pombe* gene** | **Gene product** | **References** | **Mammalian**  **orthologue** |
| --- | --- | --- | --- |
| *mid1/dmf1* | Division Mal Foutue protein | [79], [5] | Anillin/ANLN |
| *plo1* | Polo Like Kinase 1 | [78] | PLK1 |
| *pom1* | DYRK (dual specificity tyrosine phosphorylation regulated family  cell polarity kinase) | [78] | DYRK2 |
| *gef2* | Rho-guanine nucleotide exchange  factor protein | [31] | Rho-GEF |
| *blt1* | Ubiquitin domain-like protein | [34] | Not known |
| *klp8* | Kinesin-like protein | [39] | KIF13B (Kinesin family member 13B) |
| *nod1* | Medial cortical node protein | [81] | Not known |
| *rlc1* | Myosin-II regulatory light chain protein | [82] | MYL9 (Myosin  light chain 9) |
| *cdc4* | Myosin-II essential light chain protein | [83] | MYL6B (Myosin  light chain 6B) |
| *cdc12* | Cytokinetic formin | [4] | DIAPH1  (Diaphanous related formin 1) |
| *cdc15* | F-BAR domain protein | [80] | PSTPIP (Prolineserine-threonine  phosphatase interacting protein) |
| *rng2* | IQGAP domain cytoskeleton  scaffold protein | [13] | IQGAP1 (IQ GTPase activity protein 1) |
| *cdr2* | Serine/threonine protein kinase | [14] | BRSK2 (BR  serine/threonine kinase 2) |
| *clp1* | Serine/threonine protein  phosphatase | [32] | CDC14A (Cell division cycle 14A) |
| *sid2* | Serine/threonine protein kinase | [19] | STK38  (Serine/threonine kinase 38) |
| *pak1* | PAK-related kinase | [16] | PAK1 (p21 activated kinase  1) |
| *myo2* | Myosin-II heavy chain protein | [84] | MYH7B (Myosin heavy chain 7B) |
| *ark1* | Aurora kinase | [85] | AURKB (Aurora kinase B) |
| *vps4* | AAA family ATPase protein | [57] | VPS4B (Vacuolar protein sorting 4 homologue B) |

Table S2: Description of the consequences of Mid1p phosphorylation events during the *S. pombe* cell cycle.

| **Kinase** | **Phosphorylation consequences** | **Phosphorylation sites** | **References** |
| --- | --- | --- | --- |
| Plo1p | Phosphorylation of Mid1p by Plo1p triggers Mid1p release from the nucleus and promotes Mid1p association with interphase nodes leading to mitotic entry. | S15, S24, S34, S46, S62, S92, S332 | [13]; [17] |
| Cdc2p | Cdc2p-dependent phosphorylation of Mid1p by Plo1p. | T517 | [14] |
| Pak1p | Phosphorylation of Mid1p by Pak1p promotes Mid1p association with interphase nodes. | S328, S331, S403, S416, S418, S422, S425, S432 | [16] |
| Ark1p | Potential phosphorylation of Mid1p by Ark1p is required for Mid1p’s function and localization. | S523, S531 | [17] |
| Sid2p | Phosphorylation of Mid1p by Sid2p promotes Mid1p removal from the cell cortex. | S218, S227, S432, S452, S464, S531 | Willet *et al.,*  2019 |
